# Selection and evaluation of reference genes for qRT-PCR in *Inonotus obliquus*

**DOI:** 10.3389/fmicb.2025.1500043

**Published:** 2025-01-29

**Authors:** Lulu Li, Xiaofan Guo, Shouming Wang

**Affiliations:** Hubei Key Laboratory of Quality Control of Characteristic Fruits and Vegetables, College of Life Science and Technology, Hubei Engineering University, Xiaogan, China

**Keywords:** *Inonotus obliquus*, reference gene, RT-qPCR, gene expression, medicinal fungus

## Abstract

Real-time quantitative reverse transcription PCR (RT-qPCR) is one of the most effective tools for studying gene expression. *Inonotus obliquus* is a rare and edible medicinal fungus. Selecting an appropriate reference gene is crucial for researching its gene function. In this study, we selected 11 potential reference genes of *Inonotus obliquus*. Subsequently, we applied GeNorm, NormFinder, and BestKeeper algorithms to evaluate the expression stability of these 11 reference genes. Under varying carbon sources, *VPS* exhibited good stability and was suitable as an internal reference gene. Under different nitrogen sources, *RPB2* was the most stable reference gene. For varying growth factors, *PP2A* was the most stable reference gene. At different pH levels, *UBQ* showed the highest stability. Under different temperature conditions, *RPL4* was the most stable. In different strains, *RPL2* was the most stable reference gene, while *VAS* demonstrated the greatest stability across different growth stages. Overall, *VPS* was the most recommended reference gene for the entire sample set. This study provides a foundation for the further application of RT-qPCR in the gene expression analysis of *Inonotus obliquus*.

## Introduction

*Inonotus obliquus*, also known as betula antler, Ganoderma sibirica, betula mushroom, or Betula Antler, is a valuable medicinal fungus from the *Inonotus* genus within the Polyporaceae family (Song et al., 2013; Zhong et al., 2009). This fungus contains various bioactive compounds, including polysaccharides, obliquin, oxidized triterpenoids, steroids, polyphenols, and low molecular weight components (Chen et al., 2015). Among these, the polysaccharide component is considered one of the primary active substances. Numerous studies have demonstrated that the fruiting body, mycelium, and fermentation broth polysaccharides of *I. obliquus* possess pharmacological activities such as anti-tumor effects, diabetes treatment, immune regulation, antiviral properties, antioxidant activity, and anti-fatigue effects (Lu et al., 2021).

Real-time quantitative reverse transcription PCR (RT-qPCR) is a highly sensitive, specific, and accurate DNA amplification system that is widely used in gene expression studies (Chowrasia et al., 2019; Hu et al., 2022). However, the results of RT-qPCR analyses can be influenced by factors such as RNA extraction quality, reverse transcription efficiency, and amplification efficiency. In particular, selecting appropriate reference genes is crucial for the successful application of qRT-PCR in gene expression analysis (Lin et al., 2014). The use of stably expressed reference genes can significantly reduce external interference. Studies have shown that no absolutely stable reference gene exists, and the expression stability of any reference gene is subject to certain limitations. Therefore, to ensure accurate and reliable results, it is essential to screen suitable reference genes according to the specific treatment conditions used in RT-qPCR experiments (Chen et al., 2023).

## Materials and methods

### Sampling culture conditions

Except for different strain assays, *I. obliquus* JL01 was used as the material in all experiments. We set up six experiments under different culture conditions, varying the treatments while keeping the basic culture conditions constant. The nitrogen sources included peptone, soybean powder, beef paste, and yeast powder. The carbon sources included glucose, sucrose, maltose, and soluble starch. The growth factors varied among Vitamin B1 (VB_1_), Vitamin B2 (VB_2_), and Vitamin B6 (VB_6_). The culture temperatures were set at 25, 28, and 31°C. The pH values were 4, 5, 6, and 7. We used HE, JL01, and MAFF420256 strains, which are distantly related (Guo and Wang, 2020). HE originated in Russia, JL01 originated in China and MAFF420256 originated in Japan. For the culture stage assay, we first sampled the culture at 15 days. Subsequently, we collected samples every 2 days for a total of 14 sampling points. The base liquid medium consisted of the following components: glucose 2%, peptone 1%, KH_2_PO_4_ 0.3 mg%, MgSO_4_ 0.15 mg%, VB_1_ 0.1 mg%, with a pH of 5.0. After 15 days of culture, we collected samples and performed three biological replicates.

### Total RNA extraction and cDNA synthesis

For total RNA extraction from *I. obliquus* samples cultured under different treatments, we used the Ultrapure RNA kit (Cat. CW0581) and followed the kit’s specific instructions. We assessed the RNA concentration and purity using a micronucleic acid protein analyzer Nanodrop 2000 (Thermo Fisher Scientific Inc., Waltham, USA) and 1% agarose gel electrophoresis. The RNA served as the basis for subsequent cDNA synthesis. We used the Hifair III 1st Strand cDNA Synthesis Kit (Cat. 11139ES60) from Yeasen Biotechnology (Shanghai) Co., Ltd. to synthesize cDNA. Additionally, to evaluate primer amplification efficiency, we extracted RNA from *I. obliquus* cultured in the basic medium and reverse-transcribed it for use.

### Primer design and specific testing

The *I. obliquus* genome has been sequenced and its information was stored in our laboratory. In this study, we chose 11 reference genes from various functional categories: *UBQ*, *RPL2*, *PP2A*, *VAS*, *EF*, *ACTIN1*, *GAPDH*, *VPS*, *RPL4*, *RPB2*, and *TUB*, which are widely used in other edible or medicinal fungi. We designed primers for these candidate reference genes using Primer Premier 6.0, following the design principles for RT-qPCR primers. We then verified the specificity of these primers and the amplified products using ordinary PCR.

### Detection of primer amplification efficiency

We diluted cDNA obtained by reverse transcription into five gradients: 100, 10, 1, 0.1, and 0.01 ng/ul, with each gradient representing a tenfold dilution. We used these five concentrations as templates to perform RT-qPCR on the 11 candidate reference genes, with three biological replicates for each gradient. After conducting RT-qPCR, we plotted the standard curve and calculated the amplification efficiency (E) and correlation coefficient (*R*^2^) based on the slope of the primer pair’s standard curve (Wan et al., 2011).

### Quantitative real-time PCR

We used the ViiA7 real-time fluorescence quantitative PCR system to measure the expression levels of each reference gene. The reaction system consisted of 20 μL, including 10 μL of Hieff qPCR SYBR Green Master Mix (Low Rox Plus), 0.4 μL each of forward and reverse primers, 8.2 μL of ddH2O, and 1 μL of cDNA. We prepared the reaction system and performed the reaction on the ViiA7 real-time fluorescence quantitative PCR instrument. The reaction conditions were: 94°C for 5 min; followed by 40 cycles of 94°C for 10 s, 60°C for 20 s, and 72°C for 20 s. We set up three biological replicates for each sample.

### Data analysis

To identify suitable internal reference genes for *I. obliquus*, we used three common software programs: GeNorm, NormFinder, and BestKeeper, to process the data based on CT values (Supplementary Table 1). We also utilized the web tool RefFinder (Xie et al., 2023)^1^ to integrate and validate the results. Using these different data analysis methods, we evaluated the stability of the 11 candidate reference genes. We then selected the most stable reference genes across seven experimental groups, which included different nitrogen sources, carbon sources, growth factors, pH levels, temperatures, strains, and developmental stages of mycelia, all under liquid culture conditions.

## Results and discussion

### Selection and identification of the candidate reference genes

Based on previous studies (Xu et al., 2014; Yang et al., 2022), we selected 11 candidate reference genes for screening *ACTIN1*, β-tubulin (*TUB*), protein phosphatase 2A regulatory B subunit (*PP2A*), ribosomal protein L2 (*RPL2*), ribosomal protein L4 (*RPL4*), elongation factor 1-gamma (*EF*), glyceraldehyde-3-phosphate dehydrogenase (*GAPDH*), DNA-dependent RNA polymerase II second-largest subunit (*RPB2*), ubiquitin (*UBQ*), vacuolar ATP synthase (*VAS*), and vacuolar protein sorting (*VPS*).

**FIGURE 1:**
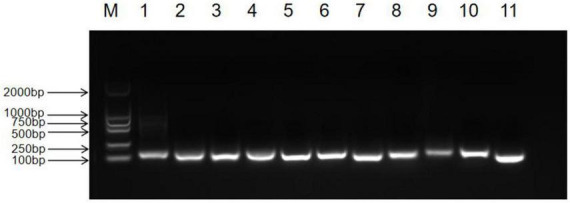
Detection of common PCR products for 11 candidate reference genes in *Inonotus obliquus* via agarose gel electrophoresis (M: DL2000 marker, 1: *UBQ*, 2: *RPL2*, 3: *PP2A*, 4: *VAS*, 5: *EF*, 6: *ACTIN1*, 7: *GAPDH*, 8: *VPS*, 9: *RPL4*, 10: *RPB2*, 11: *TUB*).

**FIGURE 2:**
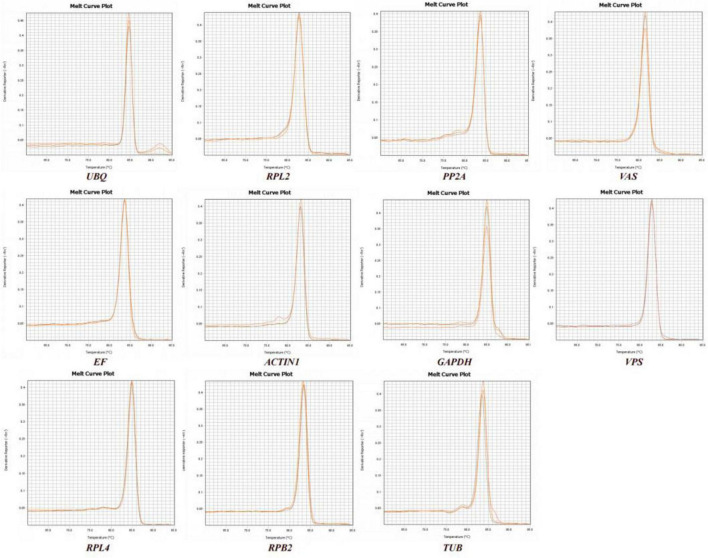
Melting curve analysis of 11 candidate reference genes.

We observed the PCR amplification product bands for these 11 candidate reference gene primers using 1% agarose gel electrophoresis. The electrophoretic images confirmed that the amplified product fragment sizes were correct, with single bands, and no non-specific amplification or primer dimerization (Figure 1). The qRT-PCR results showed that the melting curves for each candidate reference gene primer were unimodal (Figure 2). The amplification efficiency of the 11 candidate reference genes ranged from 91.29 to 110.07%, with correlation coefficients (*R*^2^) between 0.9806 and 1. Primer information is provided in Table 1.

**TABLE 1:** Amplification characteristics of 11 candidate reference gene primers.

No.	Gene name	Forward/reverse primer sequence	Tm °C	Amplication length/bp	The slope of the standard curve	*R* ^2^	RT-qPCR efficiency (%)
1	*VAS*	F:CGACTCCAAGACACAGCCATCA R:CCTGTCCACTGTCGGTCGAATC	73.2	122	−3.1624	0.9939	107.23%
2	*UBQ*	F:GGTATTCCGCCAGACCAGCA R:TCCTCCACGAAGACGCAACAC	75.5	123	−3.3367	0.9981	99.66%
3	*EF*	F:CACTCGCTGATCTCGTCATTGC R:CCTTCACCTTCGGCTGGTTGAT	76.2	132	−3.1004	0.9806	110.07%
4	*VPS*	F:TTGTCGTCACTGGCTTCGCATT R:TACACGAGACCGCCTCCGATAA	74.1	100	−3.3389	0.9897	99.30%
5	*TUB*	F:TGGTCGGTTTCGCTCCTCTCA R:CGCCATCATGTTCTTCGCATCG	75.5	104	−3.2431	0.9986	103.54%
6	*ACTIN1*	F:CCAGCCATCGTTCCTTGGACTT R:TCGTACCACCAGACAGCACAAC	74.8	125	−3.494	0.9947	93.43%
7	*RPL4*	F:CCTCCATCTTTGCCCATCCGAT R:CGACCAGAACCACGAACTTCAC	76.9	124	−3.3602	0.9814	98.44%
8	*GAPDH*	F:GCGAAGGCTGTTGGCAAGGT R:GGACGACGAGATCGACGACTGA	76.8	103	−3.2836	0.997	101.62%
9	*PP2A*	F:AGAGCGGTGGAATTGAGAGTGT R:GCGGAGTCTGGTTGTTCATTGC	75.7	108	−3.4744	0.9859	94.17%
10	*RPL2*	F:CCGAACGCCGAATGACTCTTGT R:ACCCTTCGCACCTCTCCACTTT	75.5	117	−3.5455	1	91.29%
11	*RPB2*	F:GCGAATCGGATGGCTCTACACT R:AGCAAGTCACCAGTCACGGAAT	76.2	145	−3.1253	0.9939	108.68%

### Expression profile of reference genes

**FIGURE 3:**
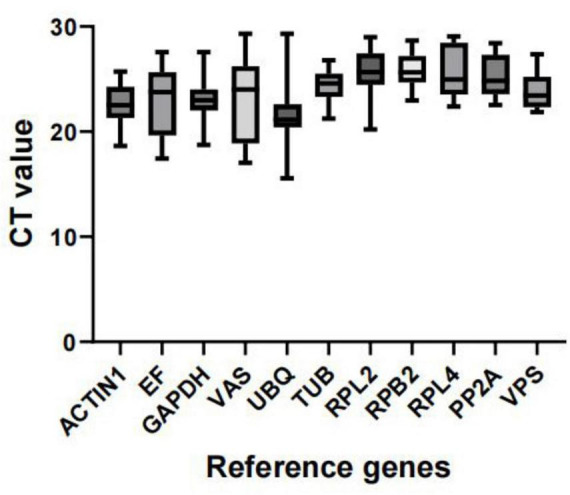
Distribution of C_t_ values for candidate reference genes. Expression data are displayed as C_t_ values for each reference gene in all samples. The line across the box depicts the median. The box indicates the 25th and 75th percentiles and the caps represent the maximum and minimum values.

We predicted the expression levels of the 11 candidate reference genes using the CT values obtained from all samples. A lower CT value indicates higher expression abundance. Figure 3 shows the distribution of CT values for these 11 reference genes across all groups. The results indicate that the CT values of the 11 reference genes under different treatments ranged from 15.55 to 29.32. Among these, the expression variation of *UBQ* in all samples was the highest (13.76). The expression variation of *VAS* was the second highest (12.288), and the expression variation of *EF* was the third highest (10.128). In contrast, *VPS* had the lowest CT value variation (5.49). Overall, other variations in transcript levels were moderate, which were sequentially sorted as *GAPDH* (8.84), *RPL2* (8.77), *ACTIN1* (7.08), *RPL4* (6.67), *PP2A* (5.87), *RPB2* (5.69), *TUB* (5.53). Based on these findings, *VPS* is the most recommended reference gene for the entire sample set (Figure 3).

### Validation of the expression stability of reference genes

To identify the most stable reference genes, we used three programs—Genorm, NormFinder, and BestKeeper—to assess gene expression stability from different perspectives. Genorm analyzed and compared the stability value (M) of reference genes across various samples to determine the most suitable candidates. A lower M value indicates higher stability, and an M value of less than 1.5 suggests that the gene can be used as a stable reference gene (Vandesompele et al., 2002).

**FIGURE 4:**
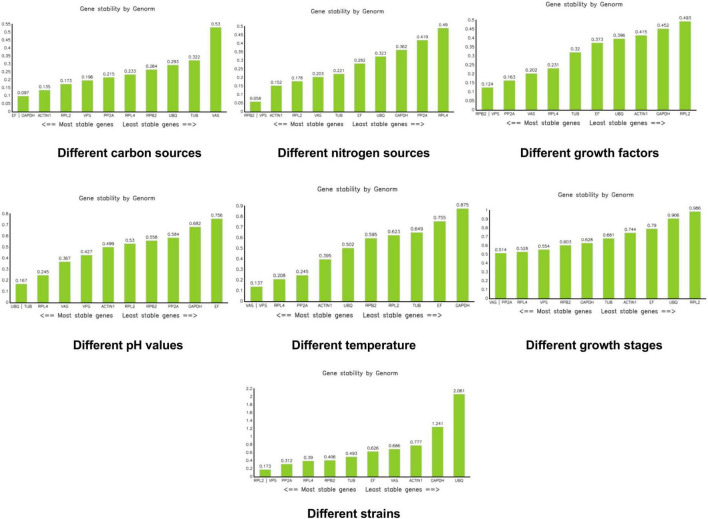
Analysis of stability values for the reference genes under different treatments using the Genorm software.

With the exception of *UBQ*, which showed an M value of 2.061 in the different strains group, all other M values across all groups were less than 1.5. Under different carbon sources, *EF* and *GAPDH* had the lowest M values. For different nitrogen sources, *RPB2* and *VPS* had the lowest M values. Similarly, *RPB2* and *VPS* were the most stable when different growth factors were added. Under varying pH conditions, *UBQ* and *TUB* showed the lowest M values. For different temperatures, *VPS* and *VAS* had the lowest M values. During different growth stages, *VAS* and *PP2A* were the most stable. Finally, under different strain conditions, *RPL2* and *VPS* had the lowest M values (Figure 4).

**FIGURE 5:**
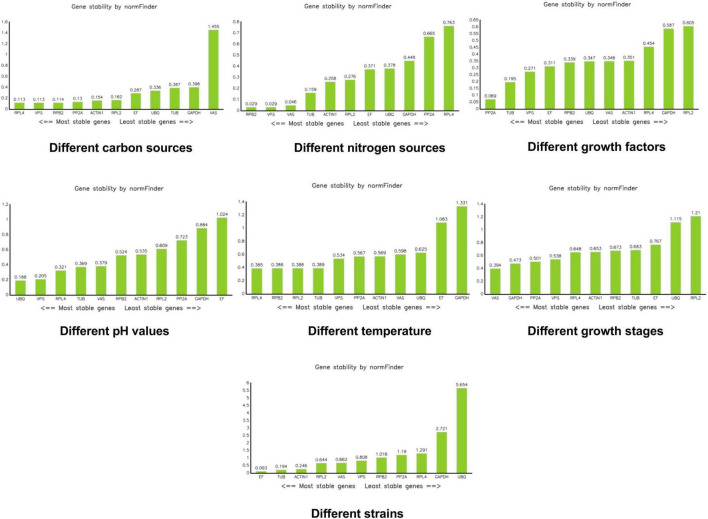
Analysis of stability values for the reference genes under different treatments using the NormFinder software.

NormFinder software calculates the expression stability value of reference genes and identifies the most suitable reference gene based on this stability value. The gene with the smallest expression stability value is considered the most suitable. NormFinder evaluates gene stability using the stability value (S); a smaller S indicates better stability of the gene (Andersen et al., 2004). According to NormFinder results, *RPL4*, *VPS*, and *RPB2* were the most stable reference genes under different carbon sources. Under different nitrogen sources, *RPB2*, *VPS*, and *VAS* were the most stable. *PP2A* was identified as the most stable reference gene under different growth factors. *UBQ* and *VPS* were the most stable reference genes under different pH conditions. *RPL4* was the most stable reference gene at different temperatures. *VAS* emerged as the most stable reference gene during different growth stages. *EF* was the most stable reference gene across the different strains (Figure 5).

**FIGURE 6:**
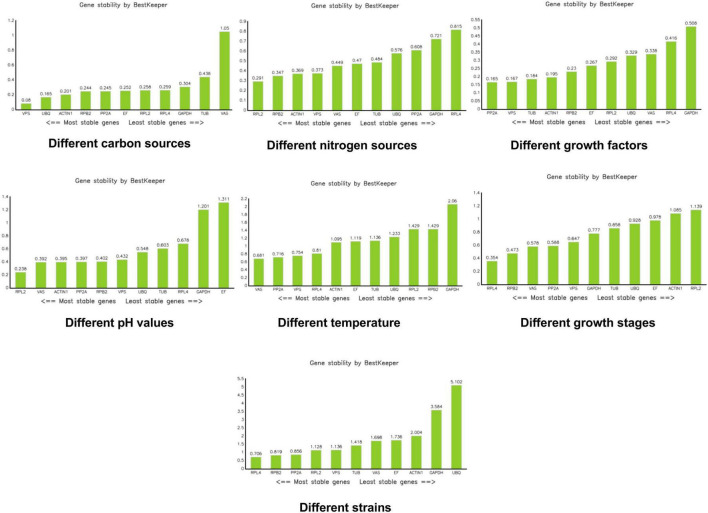
Analysis of stability values for the reference genes using the BestKeeper software.

BestKeeper assesses the stability of candidate reference genes by calculating the CT coefficient of variation (CV), standard deviation (SD), and correlation coefficient (r). Generally, a larger correlation coefficient, along with a smaller standard deviation and coefficient of variation, indicates more stable gene expression. An SD value greater than 1 suggests that the gene is unstable, while an SD value less than 1, coupled with a smaller CV, indicates higher stability of the internal reference gene (Pfaffl et al., 2004). According to BestKeeper, *VPS* was the most stable reference gene under different carbon sources. *RPL2* was the most stable reference gene under different nitrogen sources. *PP2A* and *VPS* were the most stable reference genes under different growth factors. *RPL2* was also the most stable reference gene under different pH conditions. For different temperatures, *VAS*, *PP2A*, *VPS*, and *RPL4* were considered the most stable. *RPL4* emerged as the most stable reference gene during different growth stages. Under different mycelial conditions, *RPL4*, *RPB2*, and *PP2A* were identified as the most stable reference genes (Figure 6).

Sorting by different procedures is essential for accurate evaluation. RefFinder, a comprehensive web-based tool, evaluates internal reference genes for RT-qPCR. According to RefFinder’s output, the stability of internal reference genes varied under different treatment conditions. *VPS* demonstrated good stability across different carbon source cultures. *RPB2* was the most stable reference gene under different nitrogen sources. *PP2A* was the most stable reference gene when different growth factors were used. *UBQ* showed the highest stability under various pH conditions. *RPL4* emerged as the most stable reference gene at different temperatures. *RPL2* was the most stable reference gene among different strains. *VAS* proved to be the most stable reference gene during different growth stages (Table 2).

**TABLE 2:** Comprehensive sorting of different treatment conditions.

Rank	Carbon sources		Nitrogen sources		Growth factors		pH values	
	**Gene**	**GRV**	**Gene**	**GRV**	**Gene**	**GRV**	**Gene**	**GRV**
1	*VPS*	2.11	*RPB2*	1.19	*PP2A*	1.32	*UBQ*	1.63
2	*ACTIN1*	2.59	*VPS*	2.00	*VPS*	2.06	*VAS*	3.31
3	*EF*	3.81	*RPL2*	3.46	*TUB*	2.91	*VPS*	3.31
4	*RPL4*	4.28	*VAS*	3.87	*RPB2*	3.34	*TUB*	3.36
5	*PP2A*	4.68	*ACTIN1*	3.87	*EF*	5.09	*RPL4*	4.49
6	*RPL2*	4.74	*TUB*	5.09	*VAS*	6.24	*RPL2*	4.60
7	*RPB2*	5.09	*EF*	6.74	*ACTIN1*	6.93	*ACTIN1*	5.24
8	*GAPDH*	5.18	*UBQ*	8.00	*UBQ*	7.20	*RPB2*	6.40
9	*UBQ*	6.00	*GAPDH*	9.24	*RPL4*	7.98	*PP2A*	7.35
10	*TUB*	9.74	*PP2A*	9.74	*RPL2*	9.82	*GAPDH*	10.00
11	*VAS*	11.00	*RPL4*	11.00	*GAPDH*	10.24	*EF*	11.00
**Rank**	**Temperature**				**Growth stages**		**Strains**	
	**Gene**		**GRV**		**Gene**	**GRV**	**Gene**	**GRV**
1	*RPL4*		1.86		*VAS*	1.32	*RPL2*	2.38
2	*VPS*		2.34		*PP2A*	2.21	*TUB*	2.91
3	*VAS*		2.63		*RPL4*	2.94	*VPS*	3.08
4	*PP2A*		4.28		*VPS*	3.94	*EF*	3.87
5	*RPB2*		4.53		*GAPDH*	4.12	*RPL4*	4.24
6	*RPL2*		5.42		*RPB2*	4.53	*PP2A*	4.56
7	*TUB*		5.96		*TUB*	7.24	*RPB2*	4.70
8	*ACTIN1*		6.12		*ACTIN1*	7.87	*VAS*	6.12
9	*UBQ*		7.90		*EF*	9.00	*ACTIN1*	6.64
10	*EF*		8.80		*UBQ*	9.46	*GAPDH*	10.00
11	*GAPDH*		11.00		*RPL2*	11.00	*UBQ*	11.00

GRV: geomean of ranking values.

*I. obliquus* is a rare and edible medicinal fungus due to its rich triterpenoids, polysaccharides, and sterols. It has anti-cancer, hypoglycemic, anti-oxidation, and anti-inflammation effects. However, little is known about the molecular mechanisms of its medicinal components. Here, qRT-PCR was used to study the expression of genes related to the formation of medicinal components in *I. obliquus*, which is helpful for further study of the molecular mechanism of the formation of medicinal ingredients. However, there is a lack of reports on the systematic screening of reference genes in *I. obliquus*. Different nutrients in mediums and culture conditions are very important for the formation of medicinal ingredients. Different strains at different stages of development would exhibit different medicinal ingredients. Therefore, stable reference genes are needed to explore and reveal the molecular mechanism of genes related to medicinal ingredients under these various conditions. Our experiments show that no single reference gene is suitable for all situations. However, for each culture condition, we recommend and exclude some reference genes.

Under various carbon source conditions, results obtained by the three kinds of software Genorm, Normfinder, and bestkeeper are inconsistent, but the stability of *VAS* in the bestkeeper analysis is > 1. In the carbon source experiment, we do not recommend the use of *VAS*. Results of the integrated analysis are consistent with those of bestkeeper, both of which recommending *VPS*. Moreover, its M value is 0.196 in the Genorm analysis and 0.113 in the NormFinder analysis, indicating relative stability. Therefore, we believe that *VPS* is the most stable under different carbon source conditions. Under different nitrogen source conditions, *RPB2* is the most stable in the Genorm and NormFinder analyses. In the bestkeeper analysis, the stability of *RPB2* is 0.347, which is also relatively stable. Results of the integration analysis pointed to *PPB2*, which is consistent with the results of Genorm and NormFinder. When in a medium containing different growth factors, such as VB1, VB2, and VB6, the reference genes *RPB2* and *VPS* are the most stable according to the Genorm analysis, whereas *PP2A* and *VPS* are shown to be the most stable in the NormFinder, bestkeeper, and integrated analyses. Hence, we believe *PP2A* and *VPS* are all stable, with the most stable one being *PP2A*. In various pH values, *UBQ* is recommended by Genorm, NormFinder, and integrated analyses. *GAPDH* and *EF* are the least stable, with a stability > 1. They were deemed to be unsuitable reference genes in various pH. In different temperatures, *VAS*, *VPS*, and *RPL4* all indicate good stability. However, *GAPDH*, *RPB2*, *RPL2*, *UBQ*, *TUB*, *EF*, and *ACTIN1* all indicate worse stability at > 1 according to the bestkeeper analysis. During growth development, *VAS* and *PP2A* are the best per the Genorm and integrated analyses. *RPL2* and *ACTIN1* are not recommended by according to the bestkeeper analysis. Between these different strains, *RPL2*, *VPS*, and *RPL4* all show superior stability, but *RPL2* is recommended in the first instance.

To ensure accuracy and reliability of experimental results, RT-qPCR requires the selection of stable reference genes to control for non-specific variation between samples (Ma et al., 2013). Previous studies have indicated that no single stable internal reference gene is universally suitable for all experimental conditions. Many commonly used reference genes exhibit significant variability under different treatments (Lü et al., 2018). Thus, our results systematically ascertain more appropriate reference genes for qRT-PCR analysis in *I. obliquus*. We also conclude that each setting requires a specific set of reference genes, providing a basis for the systematic study of molecular mechanisms under different experimental conditions.

## Conclusion

In this study, we employed qRT-PCR technology alongside BestKeeper, NormFinder, and GeNorm software to evaluate the expression stability of 11 candidate reference genes across different strains, growth stages, and culture conditions. Comprehensive ranking revealed the following results: under different carbon sources, *VPS* demonstrated good stability and is suitable as an internal reference gene. When varying nitrogen sources, *RPB2* proved to be the most stable reference gene. For different growth factors, *PP2A* was identified as the most stable reference gene. At different pH values, *UBQ* was the most stable reference gene. *RPL4* was the most stable reference gene at different temperatures. Among different strains, *RPL2* was the most stable reference gene. Lastly, *VAS* showed the highest stability across different growth stages. This study offers valuable insights for enhancing the accuracy of functional gene expression analysis in *I. obliquus*.

## Data Availability

The original contributions presented in this study are included in this article/Supplementary material, further inquiries can be directed to the corresponding authors.

## Footnotes

1
https://blooge.cn/reffinder/
